# Lost in transition: from medical student to clinical supervisor - a mixed-methods study

**DOI:** 10.1186/s12909-025-07313-5

**Published:** 2025-05-29

**Authors:** Capucine Delorme, Terese Stenfors, Anders Sondén

**Affiliations:** 1 Department of Clinical Science and Education, Karolinska Institutet, Södersjukhuset Stockholm, Sweden; 2 Department of Learning, Informatics, Management and Ethics, Karolinska Institutet, Stockholm, Sweden; 3 Department of Clinical Science and Education, Karolinska Institutet and Department of Surgery, Södersjukhuset, Stockholm, Sweden

**Keywords:** Supervision, Junior doctor, Clinical education, Mixed-methods study

## Abstract

**Background:**

Junior doctors play an essential role in clinical education. The transition from medical student to junior doctor involves assuming a new role as a clinical supervisor, but the preparation for this assignment is minimal and research on what the transition entails is scarce. Our study aims to explore the experiences and challenges that junior doctors face as clinical supervisors.

**Methods:**

An exploratory sequential mixed methods design, qualitative method first, was adopted. Qualitative data was collected through semi-structured individual interviews with junior doctors. Interviews were audio-recorded and transcribed verbatim. Reflexive thematic analysis was used to generate themes. Quantitative data was collected through a survey related to affections, challenges, and organizational aspects of supervision. The survey included the Positive and Negative Affects Schedule (PANAS) and self-constructed items and was analysed by calculating percentages. All data was subsequently merged and interpreted as a whole.

**Results:**

Ten junior doctors were interviewed and 89 (41%) junior doctors at two different hospitals responded to the survey. Four overarching themes were developed to describe junior doctors’ experiences as clinical supervisors: T1) *Learning to teach: Personal construct shaping supervisor development,* T2) *Learning through teaching: Dual growth from near-peer supervision,* T3) *Teaching while learning: Challenges and strategies of supervising as a novice,* T4) *Missing the manual: Organizational constrictions for efficient supervision*. The main factor contributing to junior doctors' development as supervisors was their own experiences as students (T1). Engaging in clinical supervision was rewarding for junior doctors’ learning, as it confirmed their progress in knowledge and skills and helped identify knowledge gaps (T2). However, supervising without medical expertise, formal pedagogical training, and familiarity with the clinic was a major challenge (T3). Providing effective supervision while maintaining a sustainable work-life was hindered by insufficient organizational support, and only 15% of junior doctors felt prepared to supervise (T4).

**Conclusions:**

Engaging in clinical supervision as a junior doctor can foster growth as a clinician and teacher, however, it may further increase the cognitive load that you already experience as a novice. This study highlights the affective, pedagogical, and organizational support that junior doctors need to develop as teachers.

**Supplementary Information:**

The online version contains supplementary material available at 10.1186/s12909-025-07313-5.

## Introduction

Junior doctors – referred to as ‘interns’ in many countries – play an essential role in clinical education. The transition from medical student to junior doctor involves assuming a new role as a clinical supervisor [18], but preparation for this assignment is often minimal. Clinical supervision is a complex task, and the quality of it is crucial in the formation of doctors, yet efforts to ensure effective supervision are limited [7].

Engaging junior doctors as clinical supervisors can have a positive effect on their own early professional development. This includes becoming more effective communicators, both interprofessionally and with patients. Moreover, gaining a better knowledge of pedagogical principles may help them become better learners themselves [5, 15, 29, 34, 42]. Formally developing junior doctors’ knowledge, skills, and attitudes in education may further stimulate these aspects. Although several studies confirm these positive aspects of involving junior doctors in clinical supervision, limited attention has been given to what challenges they experience when supervising students and how it affects them professionally and personally.

Current research on supervisor or teacher development often focuses on more senior clinicians, such as specialists. It shows that informal experiential learning, other life experiences, role models, formal pedagogical training and experiences as learners are important contributors to teacher or supervisor development [7, 10, 20, 24]. However, little research has explored how junior doctors learn to supervise efficiently and what support they need in that process.

Similarly, the experiences of teaching have been more explored among experienced clinicians. A frequently reported challenge faced by clinical teachers is the double burden of providing safe and high-quality patient care while simultaneously teaching students in a time-pressured environment [24, 27, 40]. Other barriers to efficient supervision include lack of protected time to supervise, insufficient encouragement and support from leadership, unclear understandings of the supervisor role and lack of supervisor training [31]. In the specific context of junior doctors, they are burdened by a stressful transition from student to practicing doctor, with newly gained responsibilities, often feeling unsupported [3] and exhausted with continuously changing workplaces [9]. How they tackle the challenge of supervising students in that position, with limited experience both as clinicians and supervisors, has not been explored in research. Professional confidence seems to affect emotional dimensions of teaching [10, 20], and as novice clinicians and supervisors, it may be particularly important.

Taken together, previous literature on what influences supervisor experience and development highlights three key areas: educational support (supervisor training, feedback on supervisor performance), organizational aspects (horizontal and vertical support for the supervisor assignment) and, less explored but perhaps more important in the context of novice supervisors, emotional aspects. This aligns with the Experience Based Learning (ExBL) framework presented by Dornan et al., who emphasizes that efficient learning in clinical workplaces depends on affective, pedagogical and organizational support [16]. Although ExBL was originally developed to understand student learning, its principles may offer useful parallels when considering how junior doctors develop into clinical supervisors.

To summarise, this study aims to explore how junior doctors experience the role as clinical supervisors and how they grow into that role, with the broader ambition to identify actionable areas of improvement to support them in their development as clinical teachers.

Research questions are:

1. How do junior doctors experience the role as clinical supervisors?
2. How do junior doctors develop as clinical supervisors?

## Methodology and methods

### Study design

An exploratory sequential mixed-methods research design, qualitative method first, was used to answer the research questions. The qualitative research was prioritized in our mixed-methods study design due to its ability to provide a richly textured account of the phenomena we were studying. Themes developed from the qualitative data were used to drive the development of a survey [12, 22]. The rationale for the use of both qualitative and quantitative data was to utilize the strengths of each method in creating a coherent picture, to complement one another in order to increase the scope, depth and consistency of the results [22]. The qualitative data was used to explore the nature of the individuals’ experiences as supervisors while the quantitative data could conceptualize findings in a matter of generalizability and magnitude of effect [13]. As such, quantitative data, secondly prioritized to qualitative findings, was used as a form of *crystallization* process, to contribute to a richly complex and multidimensional understanding of the findings [41]. In general, the interviews and the subsequent survey focused on lived experiences, perceptions and affections to address the research questions.

### Context and participants

Junior doctors doing their 18 months internship at Karolinska University Hospital and Stockholm South General Hospital in Stockholm, Sweden, between September 2023 and March 2024 were included in this study. In Sweden, a degree in medicine is obtained after 5,5 years of undergraduate studies. The latter half of the undergraduate curriculum is mainly organized around workplace learning by the rotation through a number of clinical placements of varying lengths. After graduation, medical formation proceeds with 1,5–2 years of general training (internship) before a medical license is granted and a residency program can be pursued. Doctors of all levels, including junior doctors, are engaged in clinical teaching or supervision of medical students.

### Data collection

**Fig. 1:**
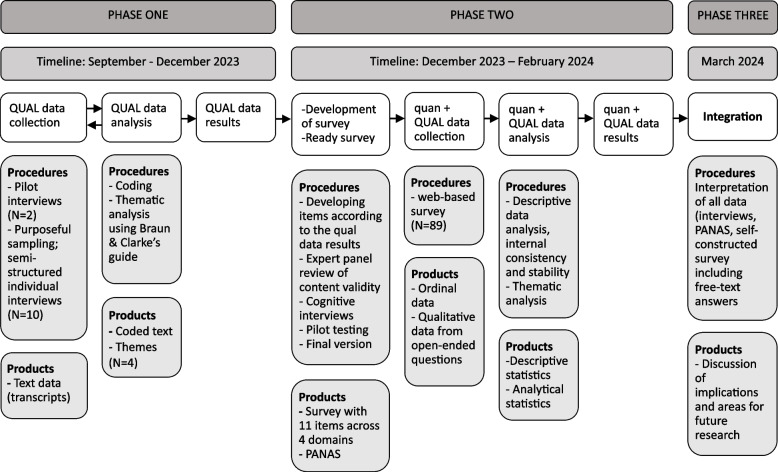
Overview of the three phases of data collection, data analysis and integration of qualitative and quantitative data. PANAS: Positive And Negative Affects Schedule

Data was collected through individual interviews and a survey. For an overview of the three phases of the study, see Fig. 1.

#### Phase 1, interviews

Semi-structured individual interviews were conducted by the first author, CD, to collect qualitative data. The preparations, interviewing process and transcription procedures were guided by the phases described by Brinkmann and Kvale [4]. The interview questions and interviewer skills were tested during two pilot interviews. An interview guide with predefined questions was developed to frame the conversations within the scope of the research questions but the semi-structured design allowed for flexibility in the arising subjects. The interview guide was inspired by a substudy lead by dr Kvernenes as part of the PROFMED (Medical students’ professional identity formation during hospital practice) project Kvernenes M, Grimstad H, Schei E, Thyness C, Dornan T, Stenfors T. Knowing me knowing you – Mentorship in Hospital Placement, Submitted. In the initial part of the interviews, participants were asked to generally describe their supervisor practice and approach. The latter, major part of the interviews then focused on how participants experienced their role as clinical supervisors in various contexts, see the English version of the interview guide under Attachments. Interviews were held face-to-face in the workplace or in an online meeting. The interviews were audio-recorded and transcribed verbatim. Participants were approached by mail. Participants were recruited through purposive sampling, targeting junior doctors in their internship period. The intended sampling frame consisted of all junior doctors undertaking their internship at Karolinska University Hospital and Stockholm South General Hospital during the study period. Inclusion criteria were defined as active clinical work during the internship, regardless of specialty rotation. Although an effort was made to achieve diversity regarding age, gender, and time of internship completion, recruitment proved challenging due to clinical workload and limited availability of potential participants. Consequently, snowball sampling was employed to supplement the participant pool, with initial participants recommending additional candidates. Ultimately, 10 participants were interviewed, which was deemed sufficient according to the concept of information power [25, 41]. An iterative cycle of data collection and analysis was performed, and after a certain number of interviews and initial thematic analysis, no new themes were identified, and the data was deemed enough to both design a survey and elicit a nuanced qualitative report.

#### Phase 2, survey

We proceeded with an online survey to further explore topics that evolved from interviews. To focus on topics relevant to our research questions, the subsequent survey was designed to address affections, challenges, and organizational aspects of supervision. All junior doctors enrolling their internship at Karolinska University Hospital and South General Hospital, (*n* = 235), were asked to participate in the survey, regardless of previous participation in the interviews in Phase 1.

1. PANAS
  We included the verified self-assessment survey PANAS (Positive and Negative Affect Schedule) [43] with the purpose of exploring the emotional aspect of supervising as a junior doctor, see the full version under Attachments. Although the PANAS was originally developed to assess affect over varying timeframes, it may also be used as a stand-alone measure to capture emotional states at a specific point in time [11, 43]. PANAS is comprised by ten items measuring positive affect and ten items measuring negative affect. The brief scale has been used extensively, and has been shown to have good internal consistency, convergency and discriminant validity [11, 43]. Response alternatives are graded on a five-point Likert Scale, varying from 5 (strongly agree) to 1 (strongly disagree), with the middle point reflecting neutrality.
2. Self-constructed survey
  After developing themes from the qualitative phase, we designed a survey intended to provide frequencies of statements that unfolded in the interviews. The items (*n* = 11, Likert-scale and open-ended questions) in the survey were constructed according to guidelines in survey design [1] and then underwent an expert validation process where experts in medical education (*N* = 6) were asked to assess the clarity and relevance of each item regarding the constructs of interest (content validity) as well as the representativeness of the survey in its entirety (face validity). This was fulfilled by the expert group filling out a content validation form and then calculating content validity index (CVI), inter-respondent agreement (IRA) and gathering free-text comments [32, 46]. Concurrently with the expert validation process, we conducted cognitive interviews (pre-planned, concurrent, verbal probing technique, audio-recorded) with representative responders (*N* = 3) to ensure that understanding and interpretation of each item was in line with the intention of the researchers [44, 45]. After modifying the survey according to results from the psycho-metric testing, we conducted a pilot testing of the final version (see Attachments for the English version) of the survey with members from the target population (*N* = 3).

### Data analysis

#### Phase 1, interviews

The transcriptions of the interviews were organized in the software program QRS Nvivo Version 13 (QSR International Pty Ltd., Doncaster, Victoria, Australia). A six-phase reflexive thematic analysis (TA) was the method of choice to explore and interpret patterns across the dataset [2]. The recursive and flexible process of TA allowed us to collect and analyse the dataset in an iterative cycle of induction and deduction to develop themes and thematic relationships. Initial themes were developed by the main researcher, discussed with all authors and then further explored and refined.

#### Phase 2, survey

The psychometric properties of the self-constructed survey were assessed by quantitative and qualitative content validity. The quantitative content validity was assessed by measuring content validity index (CVI) of both relevance and clarity of each item. The quantitative data from the surveys were converted to tables and percentages were calculated. The qualitative data from the survey were extracted and analysed with the aid of Nvivo where data codes and themes were inductively developed and compared to the interview data.

#### Phase 3, integration

The integration of qualitative and quantitative data in this mixed-methods study was achieved by the four stages described by Fetters and al, namely connecting, building, merging and embedding [17]. The two sources of data were integrated by connecting different aspects of the studied phenomena. For example, the quantitative data could describe the strength of associations while the qualitative data could more richly describe the nature of those associations [14, 17, 37].

### Translation procedures

The interviews were conducted in Swedish. Quotations included in the results section were translated into English by the first author and reviewed by all co-authors to ensure accuracy and fidelity to the original meaning. The PANAS survey was administered in Swedish using a validated translation. The self-constructed survey was originally developed in Swedish and subsequently translated into English by the authors for reporting purposes.

### Reflexivity

A reflexive practice permeated the study in its entirety, partly through a reflexive journal but generally by being mindful and observant of the influence of the researchers’ own experiences and subjectivities especially as insider researchers. The first author, CD, conducted all data collection and analysis and discussed the findings with the two other authors, AS and TS. CD and AS are both physicians and involved in clinical supervision in various ways, positioning them as insider researchers. Additionally, CD was an intern at one of the hospitals in this study and had personal acquaintance of several participants, either through the intern program or from medical school, which could potentially affect reports given by the participants. This could potentially have resulted in less exhaustive accounts if participants did not feel comfortable sharing information with the interviewer who they knew of. This risk was arguably relatively negligible, however, as we did not gather particularly sensitive data. From another view, insider perspective may serve as a strength in this study as contextual knowledge is often beneficial when formulating research questions and interpreting results. TS has a social science background with extensive experience of medical education research.

## Results

### Demographics

#### Phase 1, interviews

Ten junior doctors in various stages of their general training (internship) were included. Seven interviewees worked at Karolinska University Hospital and three at South General Hospital. Six were female and four were male. Interviews were 22–45 min long.

#### Phase 2, survey

Out of 216 junior doctors, 89 (41%) responded to the survey, 54% from Karolinska University Hospital and 46% from South General Hospital. Mean age of respondents was 32 years. 64% were women and 35% were men. Mean time of internship passed was 12,9 months (median 12 months). Out of all respondents, 97% had supervised students as junior doctors.

#### Themes

Thematic analysis of interviews resulted in four overarching themes: 1) *Learning to teach: Personal construct shaping supervisor development,* 2) *Learning through teaching: Dual growth from near-peer supervision,* 3) *Teaching while learning: Challenges and strategies of supervising as a novice,* 4) *Missing the manual: Organizational constrictions for efficient supervision*. The survey data was subsequently reviewed and was found to enrich all four themes; however, new themes were identified in the analysis. In results section below, interview and survey data are interwoven to provide a comprehensive depiction. Overall, the findings reflect the complexities of becoming a supervisor as a novice, portraying it as a rewarding yet demanding experience. Data from the interviews and the self-constructed survey are included in all themes, however, PANAS data are present in themes 2 and 4.

#### Theme 1: Learning to teach: personal construct shaping supervisor development

The main influence on junior doctors’ supervisor approach was their own prior experiences of being supervised as students. When asked to assess the distribution of different sources shaping their supervision approach, respondents estimated that approximately 54% of their supervision was based on their own experiences as students, 29% from other life experiences, 7% from education and 10% from “other” (specified in free-text comments as e.g. inherent ambitions to do good, personal characteristics or research. Stated percentages are means from each category). Supervisors that had had a distinct impact on interviewees in either a positive or negative way, i.e. role models or villain role models, majorly influenced the way they supervised students themselves. By assembling good and bad teaching experiences with supervisors, both as students and junior doctors, they constructed a supervisory technique that they felt that they themselves would have preferred as students.

*“I think I base my principles on what positive experiences of supervision I’ve had myself and maybe especially what negative experiences of supervision I’ve had.”* **R3**

*“I guess it’s like many other things in medicine, you plagiarize other supervisors that you had.”* **R4**

Supervisor development was often achieved by “learning along the way” in their clinical practice. Both interviewees and respondents in the survey also partly accredited their supervisor development to other, non-clinical experiences, such as coaching children in swimming or horse-riding, lecturing in other contexts, or even being the elder sibling of a big family. These non-clinical life experiences provided participants with skills in e.g. communication, pedagogy, or leadership that they recognized were useful in or transferable to their clinical supervisory practice. One interviewee described it as “putting on a pedagogical hat"when both coaching groups of children and supervising students.

None of the interviewees had received structured, formal feedback on their performance as supervisors from peers, superiors, or students. Similarly, in the survey, 79% disagreed or strongly disagreed to the statement “I get constructive feedback on my own performance as supervisor”. Several interviewees regularly requested verbal feedback from the students in the end of their supervisor interaction, but were concerned with the impact of the non-anonymity in the truthfulness of feedback due to hierarchical structures or students’ fear of recrimination. They often received some positive feedback which confirmed good supervision, but the feedback was still regarded as insubstantial and unable to reveal strengths and weaknesses that could help them develop as supervisors.

*“Often they say ‘thanks, it was great’, but you don’t know what you did well, you don’t know what you did badly, if something was bad, you also don’t know if the ones who didn’t say it was good, was it because they’re shy or were they unsatisfied with something…”* **R10**

#### Theme 2: Learning through teaching: dual growth from near-peer supervision

Engaging in clinical supervision of medical students was rewarding for junior doctors’ own learning path as scholars as it could both confirm a progress in knowledge and skills as well as help them identify own knowledge gaps. Several interviewees reported that supervising students enabled a sort of realization that you know more than you think, and that, although not yet medical experts in the field, you always had *some* knowledge or skills to pass to the student. This visualization and revelation of actual advancement in the life-long medical formation could improve their confidence both as scholars and clinicians.

*“I feel that I learn a lot from supervising students because they ask questions that sometimes become an affirmation that you’ve actually learned a lot along the way.”* **R5**

The positive effect on their own learning was identified as a major motivational factor for supervising students. All interviewees appreciated the challenge that supervising students induced, helping them to identify their own knowledge gaps as well as reflect on a deeper level about why and how we do things that otherwise are done routinely.

*“That’s probably the best supervising experiences, when you can kind of offer a lot but also have a student who’s active and challenge you a bit”* **R4**

Junior doctors perceived their recent experience as students as bilaterally beneficial both to their supervisory approach as well as for the student’s learning. The near-peer relationship provided them with a better understanding of the student’s needs, both regarding knowledge and generic skills. They were prone to assign students with activities that not only concerned their current learning objectives but also prepared them to their impending function as junior doctors. Several interviewees conveyed an understanding of the students’ vulnerable position and often adopted an empathic care for them, taking them under their wings.

*“Anyway, I usually try to let the students do things as much as possible. Otherwise, I feel that the contrast from being a student to your first job as a junior doctor can be quite big.”* **R2**

*“I often experience that students arrive with a name of some senior doctor who they’re supposed to go with and then you know that it is a senior doctor who won’t even look them in the eye, so you pick them up.”* **R3**

One interviewee, however, problematized the near-peer supervision approach by addressing the risk of generalizing one’s own learning preferences and experiences to students as a group, that, in some cases, social and cognitive *incongruence* may result in inefficient supervision and misunderstandings.

*“Of course it’s something that’s both for better and for worse; I think it’s positive in many aspects but you also have to remember that not everyone is like yourself. It may be easy to project your own experiences as a student to other students as a group.”* **R2**

Generally, engaging in clinical supervision added a sense of meaningfulness to their work as physicians. Being able to make a student understand or improve their clinical reasoning was identified as a key incentive to commit to supervising and aspire to become good supervisors. 82% of respondents in the survey responded “agree” or “strongly agree” to the statement “Supervising students is meaningful to me” (Table 1). Positive affections related to supervising students as junior doctors were also reflected in the ten “positive affects” measured in the Positive And Negative Affects Schedule (PANAS) that was included in the survey. Respondents generally felt *more positive affections* and *less negative affections* in their role as supervisors. The positive emotions that most respondents ranked as “quite a bit” or “extremely” include *Interested* (73%), *Enthusiastic* (57%), *Attentive* (54%) and *Active* (51%), see Table 2. These results mirror the feelings that interviewees described; that engaging in supervision of medical students was joyful (*interested, enthusiastic*) and could be challenging in a positive way, where they were kept on their toes (*attentive, active*).

*“Most importantly, I think it feels very meaningful to be part of the education, because it’s cliché but true, that the students really are the future. So I think it’s incredibly rewarding when a student says that they understood something or something makes sense, or whatever it may be.”* **R3**

**Table 1:**
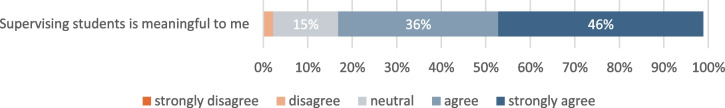
Item from self-constructed survey

**Table 2:**
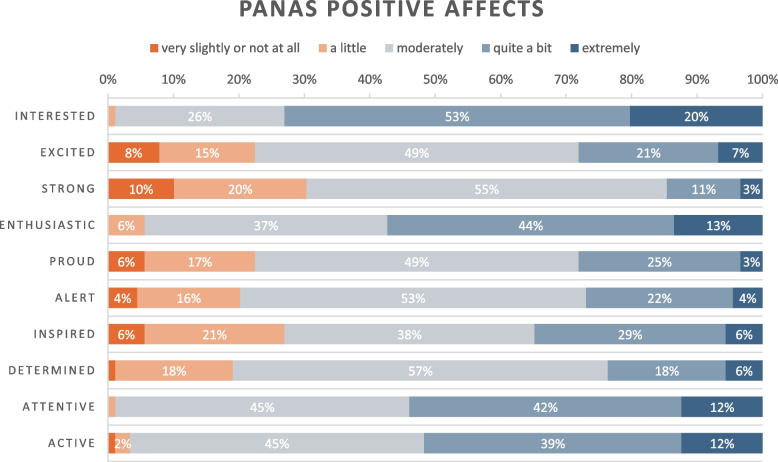
Positive And Negative Affects Schedule (PANAS), positive affections related to supervising as a junior doctor

#### Theme 3: Teaching while learning: challenges and strategies of supervising as a novice

All ten interviewees emphasized the difficulty of supervising students when you are on a learning journey of your own. They described it as a double burden to be assigned a student to supervise as a junior doctor lacking medical expertise, frequently rotating through various clinics and often being unfamiliar with practical routines at that service. Supervising students at this stage could also cause guilt and frustration to junior doctors because they perceived that the quality of the supervision was decreased due to their limited experience. These aspects were all reflected by respondents in the survey. In 69 free-text comments on what they perceive as challenging with supervising students as junior doctors, the most frequently reported themes, besides lack of time, concerned lack of clinical knowledge and experience, lack of pedagogical competence and being a newcomer yourself at the clinic.

*“It’s challenging to try to learn and pick up routines, adjust to new attendings and environments and at the same time find the time, energy and safety in that discipline to teach and supervise someone else.”* **Survey, R55**

*“I think that part of what makes it difficult to supervise as a junior doctor is that you’re supposed to supervise medical students when you’re very uncertain of the routines and practicalities where you are, in combination with having very little knowledge of what the students are supposed the learn.”* **R4**

In the PANAS part of the survey, the three negative affections that had the highest scores (that respondents graded as “quite a bit or extremely”) were *Guilty* (13%), *Nervous* (7%) and *Distressed* (3%), although most respondents (> 50%) overall rated all negative affections as low (not at all/a little), see Table 3 for details.

**Table 3:**
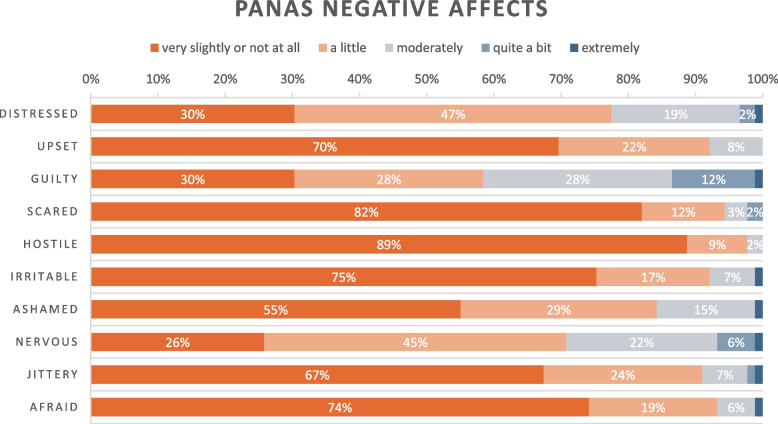
Positive And Negative Affects Schedule (PANAS), negative affections related to supervising as a junior doctor

Although supervising as a novice sometimes induced uncomfortable feelings, junior doctors had a humble attitude towards their perceived lack of expertise and fraternally used the opportunity to show students that learning is a continuous journey and that there is no shame in not knowing everything. Instead, they would “learn together” by finding answers together, which one participant described as a “learning opportunity in itself, instead of just being served the answer from someone who knows”.

*“You have to be humble and honest when you don’t know something, and I guess that’s important for them to see as well, that just because you’re a junior doctor or finish medical school you don’t know everything.”* **R8**

#### Theme 4: Missing the manual: organizational constrictions for efficient supervision

Participants generally felt unprepared for the supervisory assignment. Only 15% of respondents agreed to the statement “I often feel prepared to supervise students”. One major cause contributing to this was the lack of necessary preparatory information about the student(s) they were about to receive. 71% disagreed to receiving information in time about supervising a student and 84% disagreed to often receiving information about the students’ learning objectives (see Table 4 for details). Interviewees suggested that such information would help them both to plan the students’ placement in a student-centered, goal-directed approach, as well as plan their workday to supervise efficiently while maintaining a sustainable working life.

*“I think it’s disappointing when you arrive in the morning and don’t know beforehand, you just get a ‘oh yeah, right, there’s a student going with you today’, I think that’s disappointing. I’d like the opportunity to think about it earlier so you can plan your workday.”* **R7**

**Table 4:**
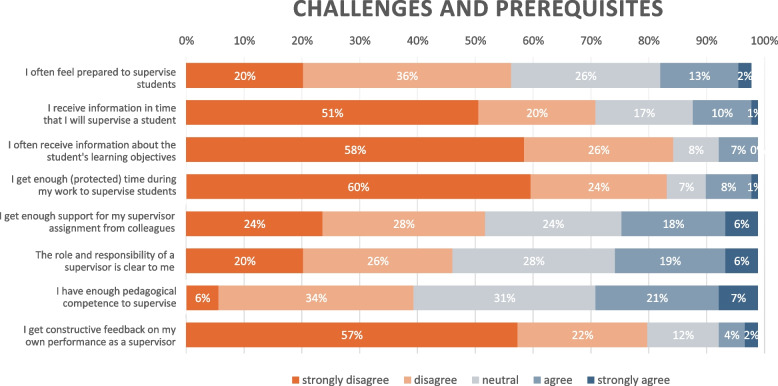
Challenges and prerequisites of supervising students as a junior doctor

In addition, only 25% of respondents agreed that the role and responsibilities of a supervisor was clear to them. Sparse instructions of the responsibilities and tasks as supervisors increased the uncertainty about the role, especially concerning assessment and dealing with underperforming or unprofessional students. This was further aggravated by a frail pedagogical structure in many clinics, where participants lacked support for their supervisor role from colleagues or the local course organizer. More than half of the respondents (52%) disagreed that they had enough support for their supervisor assignment. Collegial support, described as shared engagement for rendering meaningful clinical placements for students, (e.g. sharing interesting cases or learning activities) was appreciated but did not always occur.

*“I think the lack of structure and unclear instructions about whose responsibility it is to supervise can be the most challenging.”* **R5**

Most participants identified the lack of protected time to supervise as a major constraint to efficient supervision. In the survey, 84% disagreed to getting enough protected time during their work to supervise students. In a free-text question about challenges they face with supervising students as junior doctors, limited protected time and time management was by far the most frequent answer. Supervision often occurred as an unexpected extra workload in addition to their regular clinical work, making clinical and educational responsibilities competing tasks. However, clinical duties would always be prioritized and supervision marginalized. Interviewees described an endless conflict of having to choose between prioritizing a sustainable working life or maintaining high-quality supervision.

*“It’s almost like you have to choose between eating lunch in more than ten minutes, finish work in time or supervise. And I’ve tended to remove a lunch to have the time to supervise more, but you often end up having to choose between good working environment or supervising well.”* **R4**

*“lack of time is the biggest challenge. That you’re expected to produce the same amount of work as when you don’t have a student with you.”* **Survey, R37**

None of the interviewees had participated in a formal pedagogical or supervisor training. In the survey, only 13% of respondents had undergone any sort of supervisor training, which in free-text comments was specified as a short online introduction video common for all health care professionals in the region, a lecture about feedback, pedagogical education related to research or coaching in sports, and 28% agreed that they had enough pedagogical competence to supervise. The lack of pedagogical formation was also one of the most frequently described aspects in a free-text question about challenges with supervision as junior doctors. Several interviewees reported that the absence of formal education made them unsure of the legitimacy of their supervisory practice and that their approach was mainly based on how they preferred to be supervised as students.

*“There’s a lot of things I think are challenging, and that’s probably because I haven’t got any training for [supervising], and I’m just doing it the way I think I’d like to have it myself”* **R9**

## Discussion

In this study, we explored how junior doctors experienced the role of clinical supervisors and how they developed into this role. We found that the junior doctors were highly motivated to engage in clinical supervision of medical students, and that assuming this role had a perceived positive impact on their development as both clinicians and supervisors. However, supervising students while still being novices – both as clinician and supervisor – posed several challenges, which often evoked negative emotions and led to perceptions of inefficient supervision. Furthermore, our study shows that junior doctors primarily develop their supervisory skills from workplace experiences, initially as students being supervised and subsequently as junior doctors supervising others. Overall, this mixed-methods study provides new and comprehensive insights into clinical supervision in the context of junior doctors, allowing us to adequately address the research questions.

Experience-Based Learning (ExBL) for medical students has previously been explored by Dornan et al. [16]. In their article “*Clinical teaching for the twenty-first century*” Dornan and colleagues present a theoretical framework describing how affective, pedagogical and organisational support are critical for efficient workplace learning. Although ExBL was not used to guide the design or initial analysis of this study, our findings closely aligned with its principles. Therefore, we subsequently adopted ExBL as a useful conceptual lens for understanding how junior doctors experience and develop their supervisory role. Accordingly, we used this framework to structure the discussion below.

### Pedagogical support: Learning through experience in the absence of formal training and feedback

The main contributors to junior doctors’ development as clinical supervisors were their own experiences of being supervised and “learning by doing” while supervising others. Learning to teach through experiences as a learner and by informal experiential learning as a teacher, have both been found central in studies on teacher or supervisor development [7, 10, 20, 24]. This may be particularly important in the context of novice teachers [10]. Several factors contribute to the reliance on informal learning: lack of formal supervisor training, absence of structured feedback from students, peers or superiors on their supervisor skills, and few opportunities to collaborate with colleagues on supervision practices.

Although informal and experiential learning is valuable, it alone may not suffice for a deeper and more complex understanding of supervising and learning. Only a few participants in our study had received formal training in supervision or pedagogy, and most felt inadequately prepared for their supervisory role. Previous research has shown that lack of supervisor training results in highly variable supervision quality [19, 26, 31]. The increasing demands of excellency of both medical education and patient care, which are both intricately interwoven, require appropriately trained clinical supervisors. It is now a common view that clinical supervisors should undertake some form of training to develop their skills, attitudes and practices of a competent supervisor [6, 7, 23, 31, 40].

Two core competencies expected of clinical supervisors are solid *knowledge* *of* *basic educational principles* and *knowledge of the content being taught* [38, 39]. The view of the relationship between these two competencies varies, some describing them as intricately interlinked and others as two distinct features of a clinician. Our findings suggest that participants perceived themselves as both novice clinicians, with limited knowledge of the clinical content to be taught, and novice supervisors, with limited understanding of basic educational principles. By implementing interventions to improve the *knowledge of basic educational principles*, junior doctors might grow in their pedagogical approach and be empowered as supervisors, which, in turn, might improve their confidence as supervisors, also suggested by others [30, 42]. As described in a recent study by Noble et al. and previously by Cantillon et al., supervisory knowing in practice is highly situational and formed by cultural and social context, unique for every medical specialty [8, 27]. As junior doctors often move between specialties and cannot rely on solid clinical or educational experience in their supervisor practice, they have different needs of pedagogical support than their senior colleagues. Educational interventions to improve the knowledge of basic educational principles should therefore be formed in the context of the novice physician, to accommodate to their situational challenges and needs [8, 28].

Participants in this study rarely received structured, constructive feedback of their supervisory performance from students or peers, which is consistent with earlier findings [7, 20, 24]. Although the junior doctors indicated that they tried to obtain constructive feedback from students, the responses were often vague and unhelpful to their development as supervisors, which might be attributable to students’ fear of recrimination and an uncertainty of *what* to evaluate in supervisor performance. This highlights the need for the implementation of structural feedback systems, such as available supervisor evaluation instruments [35, 36] and a feedback culture that permeates the entire workplace to allow for increased peer evaluation, self-reflection on one’s own supervision, and institutional assessment of both individual and collective supervisor performance to improve the overall quality of clinical education [10, 31, 35, 36, 40].

### Affective support: emotional challenges in the novice supervisor role

The integration of findings from the survey and interviews provide a nuanced picture of the difficulties that junior doctors encounter, how these emotionally affect them, and to what extent. The main challenges associated with supervising in the capacity of a junior doctor appear to stem from their position as novices constantly rotating between clinical placements. This position induced feelings of insufficient medical expertise, underdeveloped professional confidence, and a persistent unfamiliarity with prevailing routines and supervision cultures within each local community of practice. This negatively impacted their role as supervisors: they expressed guilt over a perceived reduction in supervision quality, which in turn increased internal stress, and they lacked confidence due to self-doubt about their competence and skills. Although interviews revealed emotional distress among junior supervisors, the PANAS results suggest that positive emotions overruled negative emotions in the supervisor role. Nonetheless, these negative emotions had an important impact on junior doctors’ perception of their supervision, which aligns with previous research recognizing the importance of emotions in teaching [10, 20]. The deterrent effect of negative emotions on teaching has been reported before [10, 24]. In the context of junior doctors, the fear of insufficient clinical knowledge or experience as a barrier for efficient supervision has been more markedly described [21, 42], which corresponds to our findings. Emotional dimensions are further closely linked to professional confidence; therefore, strengthening junior doctors’ knowledge of educational principles may support their growth as supervisors and increase the likelihood of associating the supervisor role with positive rather than negative emotions.

In addition, colleagues and the clinical leadership can play a pivotal role in supporting and guiding junior doctors to feel safe but encouraged in their role as supervisors. In our study, junior doctors described a lack of support and engagement from leadership and colleagues in their supervisor task. Organizational culture and attitude toward supervision and teaching has been identified as important factors that permeate the entire educational climate from top to bottom and affect the commitment given by individual supervisors [7, 31]. Working in clinical settings where supervision is a recognised and valued activity has also been described as important for the development of strong supervisor identities [7, 24]. Fostering a workplace culture that values supervision and ensures that clinical educators are trained, accountable, and rewarded may improve the overall supervisor quality as well as empower the individual supervisor. For junior doctors, it may be particularly important to ensure both horizontal support (shared responsibility of the supervisor assignment with colleagues) and vertical support (someone to turn to if challenging situations occur), considering the findings from our study.

### Organizational support: structural barriers to efficient supervision

Our findings on the lack of time allocated to supervision, insufficient preparatory information about the student, and unclear description of the supervisor role and responsibilities reinforce previous reports of barriers to effective clinical supervision [31, 40]. These challenges have also been found to be particularly important in the context of junior doctors [42]. Participants in our study described a constant conflict between clinical responsibilities of patient care and supervising students efficiently, where the former was consistently prioritized. Consequently, supervisor quality and flexibility were often restricted, and/or a sustainable working life was not possible to maintain due to supervisor activities being prioritized over tasks such as administration. Our findings of clinical supervision as a marginalized task due to competing priorities align with other studies where similar results are reported in all levels of the career [24, 27, 40]. Furthermore, the tension between clinical duties and education poses a long-term risk to patient safety, as the outcome of clinical education is physicians who may not be adequately trained [33]. This underscores the need for organizational change at large, but also calls for targeted interventions to support supervisory activities in time-constrained clinical contexts. As Dornan et al. puts it, “clinicians’ leadership, which fosters three-way interactions between themselves, patients, and students, is the most important component of organizational support” [16]. Organizational support is the cornerstone of facilitating effective supervision that considers patient safety, employee health and high-quality education.

### Recommendations

We encourage faculty developers to form educational training in core supervisory skills specifically designed for junior doctors, to better prepare and pedagogically support them in their role as supervisors. Implementing interventions to improve their knowledge on educational principles may also strengthen their confidence in the role. In addition, to better evaluate and follow-up on junior doctors’ supervisor performance, they should regularly receive structured feedback. Furthermore, organizational leadership should provide necessary pre-requisites to carry out the supervisor assignment in an efficient and sustainable way.

### Methodological considerations

The quantitative part of the study included a previously validated survey, the PANAS. However, PANAS has not been validated specifically in the context of clinical supervision, and it is therefore difficult to assess how accurately it captures emotions related to supervisory experiences. Nevertheless, the emotions included in the instrument are general and should be applicable across various contexts, which is why we deemed the survey suitable for this study. The second part of the survey included a self-constructed survey, which followed guidelines in development and validation processes. The rigor of the validation process could have been further enhanced, for example, by a second validation process after revision of the survey or expanding the expert group and the pilot testing group. Due to time constraints, however, this was not feasible within the scope of this study.

It is reasonable to assume that the ones who accepted to participate in the interview and/or survey were more interested in supervision than the ones who declined, potentially resulting in a lack of insight from those less inclined to supervise in our sample. However, considering the response rate in the survey (41%), this possible nonresponse bias may be considered acceptable.

To increase transferability of our findings, the study sample was expanded through the mixed-methods design, and participants included from two different hospitals in the region. Nonetheless, the study was conducted within the specific context of junior doctors in Sweden, and certain aspects — such as the expectations placed on junior doctors, the preparation for supervisory assignments, and the structure of supervisory roles — may differ in other international contexts.

## Conclusions

This study contributes new insights into how junior doctors develop as supervisors, highlighting the central role of prior learner experiences and informal, experiential supervision. Engaging in supervision early in their careers may offer junior doctors a valuable opportunity to grow both as clinicians and as educators. However, without adequate support, supervising may increase the cognitive load that novices already experience in clinical practice. Our findings highlight the critical importance of affective, pedagogical, and organizational support in fostering the supervisory development of junior doctors. Targeted interventions that strengthen their educational knowledge, emotional resilience, and institutional support structures are needed to ensure that clinical supervision becomes a positive, sustainable, and effective component of their professional growth.

## Supplementary Information

Supplementary Material 1.

## Data Availability

Data is provided within the manuscript. The full datasets used and analyzed during the current study are available from the corresponding author on reasonable request.
