# Supplementary material for: Lost in transition: from medical student to clinical supervisor - a mixed-methods study
Source: BMC Med Educ. 2025 May 29;25:800. doi: 10.1186/s12909-025-07313-5 (PMC12123864; doi:10.1186/s12909-025-07313-5)
Supplement: Supplementary file 1 — Supplementary Material 1. [file 12909_2025_7313_MOESM1_ESM.docx]

**Attachments**

1. **Interview guide**

***Introductory questions***

When did you start your internship?

Have you worked as a junior doctor before the internship? If yes, for how long?

Have you participated in any supervisor workshop? If yes, which one?

***Questions related to supervisor approach***

Can you tell me how you supervise students?

Is there anything you feel is difficult or challenging with supervising students? If yes, what?

How do you usually give feedback to the students? Do you find this easy or difficult?

Why do you supervise the way that you do? How did you learn to supervise?

Do you have any other non-clinical experiences that have contributed to your supervisor approach? If yes, could you describe that?

Have you had any role models that inspired you in the way that you supervise?

Have you had any negative examples from being supervised yourself that affected the way that yu supervised?

***Questions related to the emotional aspect of supervision***

How do you feel when you have a student with you during your clinical work?

How does it affect your working day when you get the information that there will be students on the ward?

How does it affect your thoughts and emotions in work when you are going to supervise a student?

Can you tell me about a positive experience you have had when supervising a student? What made it positive?

Can you tell me about a negative experience you have had when supervising a student? What made it negative?

When you have a stressful day in your clinical work and get a student to supervise at the same time, how does that make you feel? How does it affect your supervision?

Have you experienced that a student that you are supervising is particularly knowledgeable in the area that you are in? How did that make you feel? How did you handle that?

Have you experienced a situation at work where you were unsure of the management of a case, whilst supervising a student? How did that feel?

Have you supervised a student that you worried about, concerning their professionalism or knowledge? How did you handle that situation? How did you feel about it?

Do you have any idea about your own performance as a supervisor? Do you receive feedback as a supervisor?

***Finishing questions***

I do not have any further questions. Is there anything you would like to add before we end the interview?

Is it alright if we get back to you if we have any further questions or need clarifications?

1. **Positive And Negative Affects Schedule (PANAS)**

This scale consists of a number of words that describe different feelings and emotions. Read each item and then mark the appropriate answer in the space next to that word. Indicate to what extent you feel this way in general when you supervise students. Use the following scale to record your answers.

|  | **Very slightly or not at all** | **A little** | **Moderately** | **Quite a bit** | **Extremely** |
| --- | --- | --- | --- | --- | --- |
| Interested |  |  |  |  |  |
| Distressed |  |  |  |  |  |
| Excited |  |  |  |  |  |
| Upset |  |  |  |  |  |
| Strong |  |  |  |  |  |
| Guilty |  |  |  |  |  |
| Scared |  |  |  |  |  |
| Hostile |  |  |  |  |  |
| Enthusiastic |  |  |  |  |  |
| Proud |  |  |  |  |  |
| Irritable |  |  |  |  |  |
| Alert |  |  |  |  |  |
| Ashamed |  |  |  |  |  |
| Inspired |  |  |  |  |  |
| Nervous |  |  |  |  |  |
| Determined |  |  |  |  |  |
| Attentive |  |  |  |  |  |
| Jittery |  |  |  |  |  |
| Active |  |  |  |  |  |
| Afraid |  |  |  |  |  |

1. **Self-constructed questionnaire**

Read each statement and indicate how strongly you agree or disagree by placing a check mark next to each statement.

|  | **Strongly disagree** | **Disagree** | **Neutral** | **Agree** | **Strongly agree** |
| --- | --- | --- | --- | --- | --- |
| 1. I often feel prepared to supervise students |  |  |  |  |  |
| 2. I receive information in time that I will supervise a student |  |  |  |  |  |
| 3. I often receive information about the student's learning objectives for the clinical placement |  |  |  |  |  |
| 4. I get enough time during my work to supervise students |  |  |  |  |  |
| 5. I get enough support for my supervisor assignment from colleagues |  |  |  |  |  |
| 6. The role and responsibility of a supervisor is clear to me |  |  |  |  |  |
| 7. I have enough pedagogical competence to do my supervisor assignment |  |  |  |  |  |
| 8. I get constructive feedback on my own performance as a supervisor |  |  |  |  |  |
| 9. Supervising students is meaningful to me |  |  |  |  |  |

| 10. If you consider what factors have shaped your way to supervise, how would you distribute, in percentage, the following (the sum should be 100%): |
| --- |
| Education in supervision and/or pedagogy ____ |
| Prior experiences as a student ____ |
| Other non-clinical life experiences ____ |
| Other ____ |
| If other, please specify here: __________________________________ |
|  |
| 11. What do you find challenging with supervising students as a junior doctor? |
